# Risk of harlequin syndrome during bi-femoral peripheral VA-ECMO: should we pay more attention to the watershed or try to change the venous cannulation site?

**DOI:** 10.1186/s13054-020-03168-y

**Published:** 2020-07-20

**Authors:** Patrick M. Honore, Leonel Barreto Gutierrez, Luc Kugener, Sebastien Redant, Rachid Attou, Andrea Gallerani, David De Bels

**Affiliations:** 1 ICU Department, Centre Hospitalier Universitaire Brugmann, Place Van Gehuchtenplein, 4, 1020 Brussels, Belgium; 2 Centre Hospitalier Universitaire Brugmann, Brussels, Belgium

We read with great interest the recent paper by Buchtele et al. who describe a new technique to detect the watershed in peripheral veno-arterial extracorporeal membrane oxygenation (VA-ECMO) [1]. We would like to make some comments. Venous cannulation for VA-ECMO is usually performed through a femoral vein and the tip of the cannula placed in the inferior caval vein or lower part of the right atrium [2]. Blood leaving the ECMO circuit is normally fully saturated with oxygen, but usually only reaches body regions supplied by the descending aorta and the distal aortic arch in peripheral cannulation, as can be shown clinically by comparing pulse oximetry saturations [2]. In a simulation study combined with a case report, changing the venous cannulation site from the inferior to the superior caval vein was shown to increase arterial saturation in the right arm from below 60% to above 80% [2]. The authors of the study concluded that venous drainage from the superior caval vein improves upper body arterial saturation during veno-arterial ECMO as compared with drainage solely from the inferior caval vein in patients with respiratory failure [2]. The main finding of their simulation study was that arterial saturations are highly dependent on the venous cannulation site during VA-ECMO in patients with severe respiratory failure [2]. Venous drainage from the superior caval vein is preferred in VA-ECMO if hypoxic respiratory failure is present [2]. This situation can be achieved easily by inserting a venous cannula through the jugular vein with a high atrial tip position or a long and wide femoral venous cannula without side holes reaching the upper part of the right atrium [2]. Our question is as follows: should we pay more attention to detecting the watershed or should we focus on applying better cannula configurations [3] in order to avoid the Harlequin syndrome as much as possible? We believe that we should apply better cannula configurations.

## Authors’ response

### Risk of harlequin syndrome during bi-femoral peripheral VA-ECMO: should we pay more attention to the watershed or try to change the venous cannulation site?—authors’ reply

Buchtele N, Staudinger T, Schörgenhofer C, Schwameis M

We want to thank Honoré et al. for their important comments concerning our recently published article [1]. The authors raised the question, whether physicians should focus their attention on detecting the watershed or emphasize venous cannula configurations, which may provide better upper body oxygenation. We completely agree that the choice of optimal cannulation sites is critical, specifically in cases with semi-elective VA-ECMO implantation. In this setting, a superior vena cava (SVC) or central arterial access might reduce or eliminate the risk of differential hypoxia.

However, our study included only patients in refractory cardiac arrest or with severe cardiogenic shock. In emergency situations with circulatory failure, bi-femoral cannulation is recommended as both femoral vessels can be approached directly and fast by vascular cutdown or modified Seldinger technique [4].

Limited neck accessibility (need for bag-valve-mask ventilation and concomitant performance of transesophageal echocardiography) and an increased bleeding risk (due to resuscitation-related coagulopathy, pre-clinical anticoagulation, or thrombolytic treatment), especially in cardiac arrest, may alter the safety and feasibility of SVC cannulation.

Upon stabilization, attention must be drawn to the occurrence of differential hypoxia in patients with concomitant pulmonary compromise. To date, monitoring for upper body hypoxia remains challenging. Pulse pressure tracing and arterial blood gas analysis from both upper extremities are commonly performed, but may lack reliability. Furthermore, cerebral oxygenation remains a blind spot and the exact location of the watershed cannot be routinely determined. In this setting, contrast-enhanced ultrasound might provide an additional, easy-to-use bedside modality helping to identify patients at risk for differential hypoxia. Recognition of the watershed may facilitate the decision-making process regarding cannulation configuration (e.g., switching to VAV configuration, SVC drainage, or central arterial cannulation) in patients that underwent acute bi-femoral cannulation.

We agree with the authors that SVC cannulation may attenuate differential hypoxia, as impressively shown in a sheep model and a septic patient, who, however, initially had to be cannulated through the femoral vein after failure of jugular vein cannulation [2, 3]. SVC cannulation may become the preferred access strategy for peripheral VA-ECMO implantation in the future, but further clinical data on safety and feasibility in emergency situations are required. A Solomonic solution comprising both strategies might be the use of long drainage cannulas for femoral cannulation passing the right atrium with their tip placed into the SVC, which has been shown to be feasible in most patients [5].

## Data Availability

Not applicable.

## Abbreviation

VA-ECMO: Veno-arterial extracorporeal membrane oxygenation
